# Differential telomerase expression in idiopathic pulmonary fibrosis and non-small cell lung cancer

**DOI:** 10.3892/or.2013.2753

**Published:** 2013-09-25

**Authors:** K.M. ANTONIOU, K.D. SAMARA, I. LASITHIOTAKI, G.A. MARGARITOPOULOS, G. SOUFLA, I. LAMBIRI, I. GIANNARAKIS, I. DROSITIS, D.A. SPANDIDOS, N.M. SIAFAKAS

**Affiliations:** 1 Department of Thoracic Medicine, University of Crete, 71110 Heraklion, Crete, Greece; 2 Laboratory of Molecular and Cellular Pulmonology, University of Crete, 71110 Heraklion, Crete, Greece; 3 Laboratory of Clinical Virology, University of Crete, 71110 Heraklion, Crete, Greece; 4 Department of Thoracic Surgery, Medical School, University of Crete, 71110 Heraklion, Crete, Greece

**Keywords:** telomerase, fibrogenesis, carcinogenesis, bronchoalveolar lavage fluid, lung tissue

## Abstract

Telomerase is a reverse transcriptase ribonucleo-protein (h-TERT) that synthesizes telomeric repeats using its RNA component (h-TERC) as a template. Telomerase dysfunction has been associated with both fibrogenesis and carcinogenesis. In this study, we aimed to evaluate the telomerase mRNA expression levels of both subunits (h-TERT and h-TERC) in lung tissue and bronchoalveolar lavage fluid (BALF) from patients with idiopathic pulmonary fibrosis (IPF) and non-small cell lung cancer (NSCLC), since there are indications of common pathogenetic pathways in these diseases. We prospectively examined lung tissue samples from 29 patients with IPF, 10 patients with NSCLC and 21 controls. Furthermore, we examined BALF samples from 31 patients with NSCLC, 23 patients with IPF and 12 control subjects. The mRNA expression for both h-TERT and h-TERC was measured by real-time RT-PCR. In the lung tissue samples, both h-TERT and h-TERC mRNA expression levels varied among the 3 groups (p=0.036 and p=0.002, respectively). h-TERT mRNA levels in the patients with IPF were lower compared with those in the controls (p=0.009) and patients with NSCLC (p=0.004). h-TERC mRNA levels in the patients with IPF were lower compared with those in the controls (p=0.0005) and patients with NSCLC (p=0.0004). In the BALF samples, h-TERT mRNA expression levels varied among the groups (p=0.012). More specifically, h-TERT mRNA levels in the patients with IPF were higher compared with those in the controls (p=0.03) and patients with NSCLC (p=0.007). The attenuation of telomerase gene expression in IPF in comparison to lung cancer suggests a differential role of this regulatory gene in fibrogenesis and carcinogenesis. Further functional studies are required in order to further elucidate the role of telomerase in these devastating diseases.

## Introduction

Idiopathic pulmonary fibrosis (IPF), accounting for >50% of cases with idiopathic interstitial pneumonia (IIP), is a progressive, lethal disease, whose etiology remains enigmatic (1,2). The majority of IPF cases are sporadic, while 2–20% of cases are familial, inherited in an autosomal dominant pattern with incomplete penetration (2). The current hypothesis supports the notion that IPF is not an inflammatory disorder, but a complex process characterized by abnormal pneumocyte apoptosis and the profound disruption of the renewal of the alveolar epithelium, making it, at least in many aspects, quite similar to malignant lung disease (2–4).

IPF and lung cancer have several striking similarities. Both are fatal lung diseases, whose main event is aberrant cell proliferation and they share a number of pathogenetic pathways (4–6). Genetic alterations, response to growth and inhibitory signals, resistance to apoptosis, myofibroblast origin and behavior, altered cellular communication and intracellular signaling pathways are all fundamental pathogenic hallmarks of both IPF and cancer (4–6). Furthermore, both diseases are characterized by the lack of effective treatment and a poor survival rate, a combination that underlines the need for further research for novel information and perspectives (2,4–6).

Mutations of various genes have emerged in search of the etiology of both diseases, such as the ‘aging’ gene, telomerase (3,4). Telomeres are repetitive DNA sequences at the end of chromosomes, which protect them from degradation, irregular recombination and end-to-end fusions (3). Telomeres decrease in length with every cell division until they reach a critically short size and signal the arrest of cell proliferation, senescence and apoptosis (5). The active telomerase ribonucleoprotein complex contains 3 subunits: the telomerase reverse transcriptase (h-TERT), the RNA subunit (h-TERC) and dyskerin (6).

The catalytic activity of this enzyme resides in the h-TERT component, and thus the regulation of h-TERT mRNA expression seems to be the most important step for telomerase activation (7). Although h-TERT seems to be the key component in telomerase regulation and telomere synthesis, h-TERC is also required to maintain cell growth, particularly when h-TERT is overexpressed (8). It appears that h-TERC may play a stabilizing role in the telomerase complex. h-TERT is highly expressed in germ cells, cells with proliferative potential and in immortalized cancer cells (9–11), although in most other cells, telomerase activity is restricted in humans (12). In addition, in IPF, 25% of individuals who have either familial or sporadic pulmonary fibrosis, without h-TERT or h-TERC mutation, have shorter telomeres in their circulating leukocytes (2).

Since there are indications of common pathogenetic pathways between IPF and lung cancer, in this study, we aimed to evaluate the expression levels of both telomerase subunits (h-TERT and h-TERC) in lung tissue and bronchoalveolar lavage fluid (BALF) from patients with IPF and non-small cell lung cancer (NSCLC).

## Patients and methods

### Patients

Consecutive patients with IPF and NSCLC from the Department of Thoracic Medicine, University Hospital of Heraklion, Crete, Greece were enrolled in this study. The diagnosis for IPF was based on internationally accepted clinical and imaging criteria, using video-assisted thoracoscopic surgery (VATS), where needed (13). All patients were sporadic IPF cases. The diagnosis for NSCLC was based on histopathological criteria from bronchial biopsies and cytology from bronchial washings. The patients included in this study were classified as NSCLC according to the WHO criteria (1997). All IPF and NSCLC patients were newly diagnosed and treatment naive at the time of either bronchoscopy or surgery.

The lung tissue samples were obtained from 29 patients with IPF, 10 patients with NSCLC and 21 controls. NSCLC samples were obtained from sections of the lung with verified positive histology. Control lung tissue specimens were obtained from patients undergoing lobectomy or pneumonectomy for bronchogenic carcinoma, at a macroscopically healthy site distant from the malignancy. The samples were further verified histologically as free of malignancy before being classified as control samples, as previously described by ours and other groups (14,15). The BALF samples were obtained from 23 patients with IPF, 31 patients with NSCLC and 12 controls. Control subjects were patients undergoing bronchoscopy for the investigation of haemoptysis, without any pulmonary comorbidities and with normal bronchoscopic findings and cytology results. Subjects who had experienced respiratory infections during the 6 weeks prior to bronchoscopy or surgery were excluded from this study. All patients were of comparable age. The patients with NSCLC and the control subjects exhibited near normal pulmonary function tests, whereas the patients with IPF had a mild restriction with decreased pulmonary volumes. Demographics and pulmonary function tests of the patients and controls are presented in Table I.

### Ethics statement

Informed consent was obtained from all patients and control subjects who participated in this study. The study protocol was approved by the Ethics Committee of the University Hospital of Heraklion.

### Biological samples and processing

BALF was obtained from all patients at room temperature as previously described (16). After filtering through a sterile gauze (Thompson, Ontario, Canada) to remove debris, BALF from each patient was centrifuged at 400 × g for 15 min at 4°C and the supernatant and pellet were stored at −80°C. Lung tissue biopsy specimens were obtained at room temperature, immediately frozen in liquid nitrogen and stored at −80°C.

### Gene expression

BALF pellets and homogenized lung tissue samples were processed using the TRIzol reagent (Invitrogen, Carlsbad, CA, USA) protocol for total RNA extraction according to the manufacturer’s instructions. RNA concentration and purity were evaluated using a spectrophotometer. Aliquots of RNA were stored at −80°C until use. cDNA from each sample was derived by reverse transcription of 2 μg of total RNA using the AffinityScript™ Multi Temperature cDNA synthesis kit, (Stratagene, La Jolla, CA, USA). Random hexamers were used as amplification primers. To remove the RNA template, cDNA was incubated with *E. coli* RNaseH and stored at −20°C until use.

Transcript levels of h-TERT, h-TERC and transforming growth factor (TGF)-β were determined using the Mx3000P Real-Time PCR system (Stratagene) and SYBR-Green I Master Mix (Stratagene) according to the manufacturer’s instructions, as previously described (17–19). All primers were designed to span at least one intron in order to avoid amplification of contaminating genomic DNA. β-globin was used as an internal control to normalize mRNA expression levels, as previously described by our study group (17–19). To verify the results of the melt curve analysis, PCR products were analyzed by electrophoresis on 2% agarose gels stained with ethidium bromide and photographed on a UV light transilluminator. Primer sequences and annealing temperatures for all the genes analyzed, as well as for β-globin are presented in Table II.

### Statistical analysis

The one sample Kolmogorov-Smirnov test was employed to assess normality. Data were compared using Kruskal-Wallis ANOVA with the Mann-Whitney test for post-hoc comparisons. The percentages of patients expressing hTERT, h-TERC and TGF-β were compared using the χ^2^ test. Values are expressed as the median (lower-upper quartiles) and a value of p<0.05 was considered to indicate a statistically significant difference. Linear regression analysis (Spearman’s rank correlation coefficient) was used to assess TGF-β expression in the lung tissue. Statistical calculations were performed using Statistica 7 software (StatSoft, Tulsa, OK, USA).

## Results

### Gene expression in lung tissue

**Figure 1:**
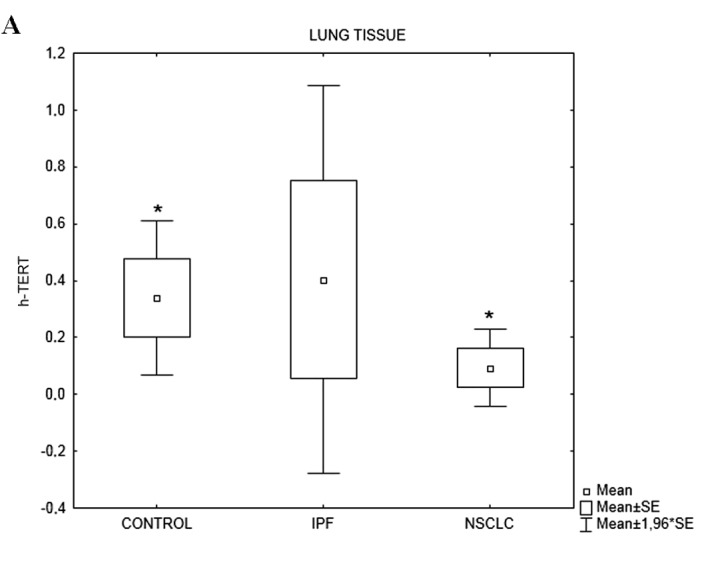

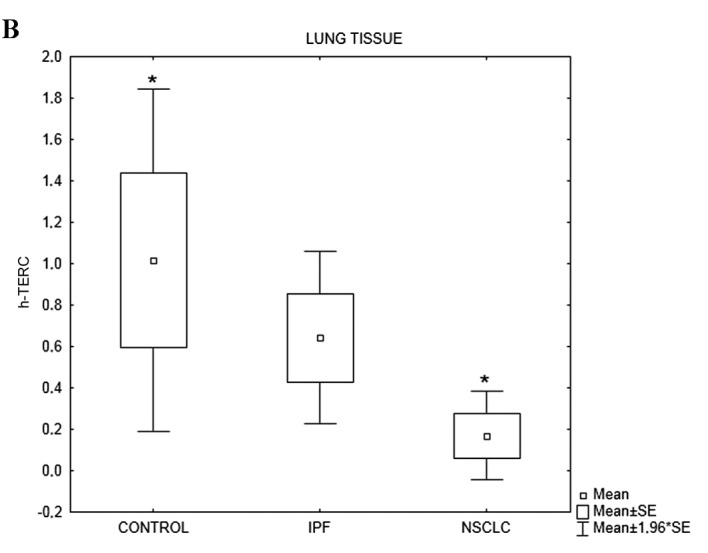
(A) h-TERT and (B) h-TERC relative mRNA expression in the lung tissue samples from the control subjects (parenchyma) and patients with IPF and NSCLC (tumor samples). ^*^p<0.05 vs. IPF. NSCLC, non-small cell lung cancer; IFP, idiopathic pulmonary fibrosis.

As regards the h-TERT subunit, the telomerase gene was expressed in 52.4% of the controls, 13.8% of the patients with IPF and 60% of the NSCLC population (Table III). As regards the h-TERC subunit, the telomerase gene was expressed in a significantly lower number of patients with IPF compared with the controls and patients with NSCLC (Table III).

h-TERT mRNA expression levels differed among the 3 groups (p=0.036). h-TERT mRNA levels in the patients with IPF were significantly lower compared with those in the controls (p=0.009) and patients with NSCLC (p=0.004). The majority of subjects in all 3 groups expressed h-TERC mRNA (Table IVA). h-TERC mRNA expression levels differed among the 3 groups (p=0.002). Again, h-TERC mRNA levels in the patients with IPF were significantly lower compared with those in the controls (p=0.0005) and patients with NSCLC (p=0.0004) (Table IVA, Fig. 1).

TGF-β mRNA expression levels differed among the 3 groups (p<0.0001). TGF-β mRNA levels in the patients with IPF were significantly higher compared with those in the controls and patients with NSCLC (Table IVA). Using linear regression analysis (Spearman’s rank correlation coefficient), we assessed that TGF-β expression in the lung tissue positively correlated with h-TERT and h-TERC expression in a very small percentage of patients with IPF and NSCLC (Table V).

### Gene expression in BALF

**Figure 2:**
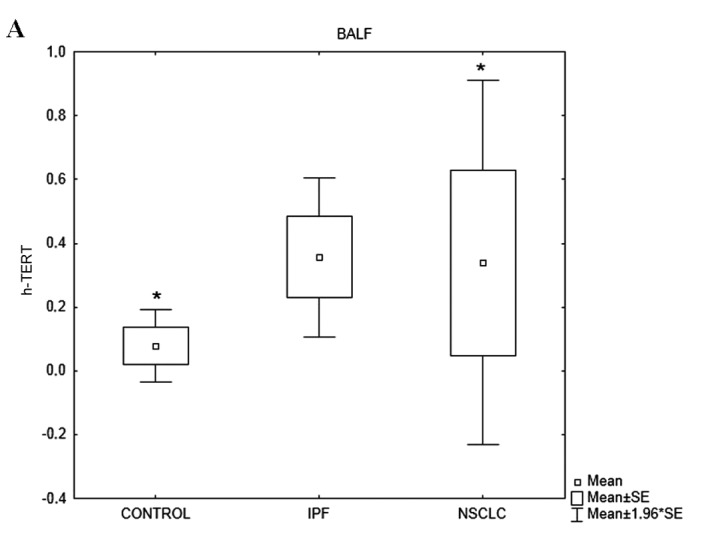

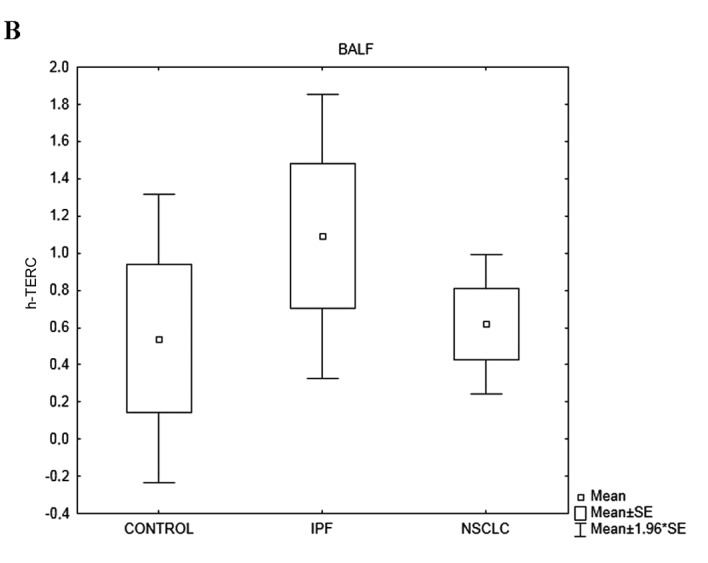
(A) h-TERT and (B) h-TERC relative mRNA expression in the BALF samples from the control subjects and patients with IPF and NSCLC. ^*^p<0.05 vs. IPF. NSCLC, non-small cell lung cancer; IFP, idiopathic pulmonary fibrosis; BALF, bronchoalveolar lavage fluid.

The percentage of the patients with NSCLC and control subjects expressing h-TERT was lower than that of patients with IPF (p<0.05, Table III). The percentages of h-TERC mRNA expression did not differ significantly among the groups (Table III).

h-TERT mRNA expression levels varied among the groups (p=0.012) (Table IVB). More specifically, h-TERT mRNA levels in the patients with IPF were significantly higher compared with those in the controls (p=0.03) and patients with NSCLC (p=0.007). h-TERC mRNA expression levels varied among the 3 groups (p=0.07). Post-hoc analysis revealed that h-TERC mRNA levels in the patients with IPF tended to be higher compared with those in the control subjects (p=0.08) (Table IVB, Fig. 2).

**Figure 3:**
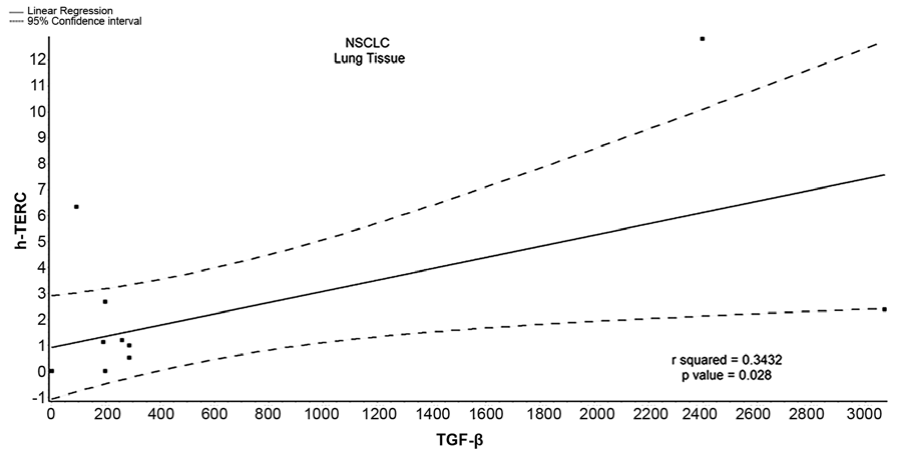
Correlation of h-TERC relative mRNA expression with TGF-β relative mRNA expression in lung tissue samples from patinets with NSCLC (tumor samples) (r2=0.343, p=0.028). NSCLC, non-small cell lung cancer.

TGF-β mRNA expression levels differed among the 3 groups (p<0.0001) (Table IVB). Using linear regression analysis (Spearman’s rank correlation coefficient), we assessed that TGF-β expression in the BALF samples negatively correlated with h-TERT and h-TERC expression in a very small percentage of patients with IPF (Table V). On the contrary, TGF-β expression positively correlated with h-TERC in the BALF samples from patients with NSCLC with a statistically significant p-value (r^2^=0.343, p=0.028), while the positive correlation between TGF-β expression and h-TERT was very weak and statistically insignificant (Table V, Fig. 3).

## Discussion

The main finding of this study is the attenuated expression of both telomerase subunits measured in lung tissue obtained from patients with IPF, compared with lung tissue obtained from patients with NSCLC and control subjects. To our knowledge, this is the first time that h-TERT/h-TERC expression levels have been measured in human lung tissue and BALF.

Fibrogenesis and carcinogenesis are attractive research topics, as although therapeutic efforts have increased over the past decade, few advances have been made (4–6,20). Certain studies have suggested a link between IPF and lung cancer through different pathogenetic mechanisms, such as viral implications, inflammation, coagulation, dysregulated apoptosis, focal hypoxia, activation of oncogenes, genetics and the accumulation of myofibroblasts, as well as extracellular matrix accumulation (21,22). However, diversities in the expression of molecular pathways have also been recently described by ours (14,23) and other study groups (4–6). Previous studies have demonstrated that mutations in telomerase h-TERT and h-TERC genes cause a shortening of telomere lengths, and account for approximately 10% of familial IPF cases (24–26).

In this study, h-TERT mRNA expression was downregulated in the lung tissue samples obtained from patients with IPF, which is the most novel finding of our study. Our study was not designed to unravel the mechanism of h-TERT downregulation in this disease. Nevertheless, some speculations are worth pursuing. Decreased h-TERT expression levels may represent decreased h-TERT mRNA transcription due to downregulation by a variety of factors, such as p53, TGF-β, Wilms tumor-1 (Wt-1) and murine double minute 2 (Mdm2) (27), which have been implicated in the pathogenesis of IPF. These factors have been shown to negatively regulate h-TERT, thus limiting telomerase activation (28). However, it should be acknowledged that h-TERT activity may also be regulated by alternative splicing and post-translational modifications (27). As h-TERT is the catalytic subunit of the telomerase complex it can be hypothesized that the attenuated expression also involves the downregulation of telomerase activity. Another possible explanation for the attenuated h-TERT expression measured in our patients compared with the healthy controls is the existence of mutations in the h-TERT gene in patients with IPF, which would inhibit telomerase activity. However, telomerase mutations in sporadic IPF cases are rare, detected in only 1 out of 100 patients in the general population (29).

In contrast to the lung tissue samples, h-TERT expression was augmented in the BALF samples compared with the healthy controls. This suggests that BALF is not representative of telomerase expression in tissue and cannot be recommended as surrogate material of telomerase expression determination in patients with IPF. The reason for the increased expression in BALF is not clear and cannot be addressed by the results of our study. It is highly possible that macrophages and neutrophils which constitute the major cell subpopulations in BALF from patients with IPF (30) exhibit increased h-TERT expression, which is in accordance with previous results from our group, where bone marrow-derived mesenchymal stem cells from patients with IPF showed a trend for increased h-TERT expression compared with the healthy controls (31). h-TERC mRNA expression levels exhibited the same pattern as the h-TERT levels in patients with IPF.

The assessment of h-TERT/h-TERC mRNA expression in lung tissue and BALF from patients with NSCLC revealed a profile similar to that of the control group for both subunits. In the lung tissue samples, a trend for increased h-TERC expression was observed compared with the controls, although statistical significance was not achieved. However, when comparing NSCLC with IPF, significant differences were observed. More explicitly, both h-TERT and h-TERC expression levels were significantly decreased in the tissue samples from patients with IPF compared with the patients with NSCLC, suggesting that telomerase genes play a differential role in fibrogenesis and carcinogenesis. An analysis of the BALF samples revealed increased h-TERT expression levels in patients with IPF compared with patients with NSCLC, again suggesting differences in the telomerase pathway between these two diseases.

This study further explored the correlation between a known fibrogenic gene, namely TGF-β, and h-TERT, as well as h-TERC gene expression. The results revealed no significant correlation between fibrotic gene expression, represented by TGF-β, and telomerase gene expression in the IPF samples. Of note, TGF-β expression in the lung tissue samples from patients with NSCLC was found to significantly correlate with h-TERC gene expression. Alveolar epithelial cells exposed to TGF-β have been shown to gradually lose epithelial markers, such as cytokeratin, and acquire specific mesenchymal markers, such as α smooth muscle actin (α-SMA), vimentin and type I collagen (6). In pulmonary fibrosis, epithelial cells surrounding fibroblast foci express both epithelial and mesenchymal markers, suggesting that epithelial-mesenchymal transition (EMT) occurs in those areas of lung tissue, supporting an active role for EMT in lung fibrogenesis. As EMT is a form of metaplasia, it is also involved in the early steps of carcinogenesis and cancer cell invasion (6). Recent data from an *in vitro* study indicate that hTERT overexpression promotes the EMT of cancer cells, thereby contributing to lung cancer progression through a TGF-β- and β-catenin-mediated pathway (32).

### Implications and limitations

In this study, we measured h-TERT/h-TERC expression in both BALF and lung tissue samples. Thus, although we did not determine the cells of origin, combining both tissue and lavage fluid allowed us to provide complementary information that elucidates the expression pattern of telomerase in these diseases. Possible cells of origin of the h-TERT/h-TERC expression from our BALF and lung tissue samples are alveolar epithelial cells (30), alveolar macrophages and leukocytes infiltrating within the lung vessels or the interstitium. Our group has previously demonstrted that BALF epithelial cells exhibit genetic instability [microsatellite instability (MSI)/loss of heterozygosity (LOH)] compared with leukocytes/macrophages (33), which suggests that the reduced telomerase expression in epithelial cells may contribute to the attenuated telomerase expression in the lungs of patients with IPF. One further limitation of the present study was that we did not confirm these findings on the protein level in order to exclude the possibility of non-coding RNA at the post-transcriptional level. Further studies are required in order to clarify the cell(s) of origin of telomerase expression in the healthy and diseased human lung. We did not measure telomere length, as we did not study separate cell subpopulations of BALF or lung tissue samples, which is a limitation of our study. Certain data indicate that shorter telomeres in peripheral blood lymphocytes positively correlate with telomere length measured in alveolar epithelial cells from the same individuals (29). Yet, it is not certain that the degree of telomere shortening observed in circulating leukocytes is representative of the telomere length of resident lung cells of the same subjects (34).

In conclusion, in the present study, we demonstrate that both h-TERT and h-TERC mRNA expression is downregulated in lung tissue from patients with IPF compared with healthy controls. The activation of attenuated telomerase genes in IPF has been implicated as a potential therapeutic strategy (31,35,36). Moreover, h-TERT and h-TERC expression levels were found to be significantly decreased in tissue samples from patients with IPF compared with the patients with NSCLC. These results do not provide support for a common pathway hypothesis concerning the telomerase pathway, but rather reveal distinct telomerase activation profiles between NSCLC and IPF. However, further studies are required to evaluate telomere length in both diseases.

## Figures and Tables

**Table I:** Clinical characteristics and pulmonary function tests of all patients studied.

A, Lung tissue samples				

Characteristics	Controls	IPF	NSCLC	p-value
No.	21	29	10	
Age^a^	63.41±4.12	67.8±3.93	59.38±6.81	p1, p2, p3=NS
Gender (M/F)	17/4	21/8	9/1	
Non-smoker	9	13	0	
Smokers	6	5	8	
Ex-smokers	6	11	2	
FEV1^a^	85.31±8.49	75.64±4.23	83.78±5.74	p1, p2, p3=NS
FVC^a^	93.22±7.50	74.85±3.30	92.19±8.25	p1<0.05, p2, p3=NS
FEV1/FVC^a^	73.33±4.29	82.14±1.85	71.89±4.91	p1, p3<0.05, p2=NS
DLCO^a^	74.43±11.37	50.73±4.30	-	p1<0.05

B, BALF samples				

Characteristics	Controls	IPF	NSCLC	p-value

No.	12	23	31	
Age^a^	61.75±3.51	69.17±1.23	66.62±1.74	p1, p2, p3=NS
Gender (M/F)	10/2	18/5	27/4	
Non-smoker	5	9	2	
Smokers	4	3	18	
Ex-smokers	3	11	11	
FEV1^a^	86.78±8.71	77.88±3.45	82.34±4.71	p1, p2, p3=NS
FVC^a^	91.88±6.90	71.68±4.52	91.13±8.92	p1, p3<0.05, p2=NS
FEV1/FVC^a^	75.41±5.19	85.31±1.79	72.14±5.23	p1, p3<0.05, p2=NS
DLCO^a^	72.28±9.71	52.69±4.13	-	p1<0.05

Values are expressed as the means ± SEM (standard error of the mean).

a t-test; p<0.05 was considered to indicate a statistically significant difference.

NS, not significant. p1, IPF vs. controls; p2, NSCLC vs. controls; p3, IPF vs. NSCLC. NSCLC, non-small cell lung cancer; IFP, idiopathic pulmonary fibrosis; BALF, bronchoalveolar lavage fluid; M, male; F, female; FEV1, forced expiratory volume in 1 sec; FVC, forced vital capacity; DLCO, diffusing capacity for carbon monoxide.

**Table II:** Primer sequences used for real-time RT-PCR.

Gene	Primer pair sequence (5′→3′)	Annealing temperature (°C)
TGF-β	Forward AAGGACCTCGGCTGGAAGTGC Reverse CCGGGTTATGCTGGTTGTA	62
h-TERT	Forward TGACACCTCACCTCACCCAC Reverse CACTGTCTTCCGCAAGTTCAC	51
h-TERC	Forward GCCTGCCGCCTTCCACCGTTCATT Reverse GACTCGCTCCGTTCCTCTTCCTG	59
β-globin	Forward GCTTCTGACACAACTGTGTTCACTAGC Reverse CACCAACTTCATCCACGTTCACC	58

**Table III:** Percentage of subjects expressing h-TERT and h-TERC mRNA in lung tissue and BALF samples.

A, Lung tissue samples				

	Control (n=21)	IPF (n=29)	NSCLC (n=10)	p-value^a^
h-TERT	52.4%	13.8%	60%	p1=0.003 p2=NS p3=0.004
h-TERC	61.9%	17.3%	90%	p1=0.001 p2=NS p3=0.0001

B, BALF samples				

	Control (n=12)	IPF (n=23)	NSCLC (n=31)	p-value^a^

h-TERT	33%	65.2%	25.8%	p1=0.07 p2=NS p3=0.004
h-TERC	50%	73.9%	59.1%	p1, p2, p3=NS

a χ^2^ test, p<0.05 was considered to indicate a statistically significant difference.

NS, not significant p1, IPF vs. controls; p2, NSCLC vs. controls; p3, IPF vs. NSCLC. NSCLC, non-small cell lung cancer; IFP, idiopathic pulmonary fibrosis; BALF, bronchoalveolar lavage fluid.

**Table IV:** h-TERT and h-TERC relative mRNA expression levels in lung tissue and BALF samples from patients with IPF and NSCLC and the control subjects.

A, Lung tissue samples				

	Control (n=21)	IPF (n=29)	NSCLC (n=10)	p-value
h-TERT	0.01 [0–0.35]	0.00 [0–0]	0.695 [0–2.34]	0.036
h-TERC	1.12 [0–2.66]	0.00 [0–0]	0.97 [0.68–2.88]	0.002
TGF-β	0.7390 [0.1280–3.220]	194 [0.0015–3070]	0.00 [0.00–0.03]	<0.0001

B, BALF samples				

	Control (n=12)	IPF (n=23)	NSCLC (n=31)	p-value

h-TERT	0.00 [0–0.02]	0.09 [0–0.23]	0.00 [0–0.01]	0.012
h-TERC	0.005 [0–0.385]	0.44 [0–1.21]	0.06 [0–0.73]	0.07
TGF-β	100.3 [1.51–533.6]	199.2 [1.873–11200000]	0.0020 [0–2.7933]	<0.0001

Data are presented as the median (lower-upper quartiles) and a value of p<0.05 was considered to indicate a statistically significant difference, Kruskal-Wallis test. NSCLC, non-small cell lung cancer; IFP, idiopathic pulmonary fibrosis; BALF, bronchoalveolar lavage fluid.

**Table V:** Correlation of h-TERT and h-TERC relative mRNA expression with TGF-β relative mRNA expression in patients with IPF and NSCLC.

	Group	
	
Telomerase gene per biological sample	IPF	NSCLC
TGF-β relative mRNA expression in BALF correlated with h-TERT	r^2^=0.015, r=0.3012, p=NS	r^2^=0.006, r=0.055, p=NS
TGF-β relative mRNA expression in BALF correlated with h-TERC	r^2^=0.022, r=0.070, p=NS	r^2^=0.016, r=0.126, p=NS
TGF-β relative mRNA expression in lung tissue correlated with h-TERT	r^2^=0.106, r=−0.074, p=NS	r^2^=0.006, r=0.077, p=NS
TGF-β relative mRNA expression in lung tissue correlated with h-TERC	r^2^=0.089, r=−0.065, p=NS	r^2^=0.343, r=0.585, p=0.028

NSCLC, non-small cell lung cancer; IFP, idiopathic pulmonary fibrosis. BALF, bronchoalveolar lavage fluid.
